# Unveiling oxygen vacancy impact on lizardite thermo and mechanical properties

**DOI:** 10.1038/s41598-023-44424-9

**Published:** 2023-10-11

**Authors:** H. Pecinatto, Celso R. C. Rêgo, W. Wenzel, C. A. Frota, B. M. S. Perrone, Maurício J. Piotrowski, Diego Guedes-Sobrinho, Alexandre C. Dias, Cicero Mota, M. S. S. Gusmão, H. O. Frota

**Affiliations:** 1 PPG-FIS, Federal University of Amazonas, Manaus, AM Brazil; 2 Karlsruhe Institute of Technology (KIT), Institute of Nanotechnology Hermann-von-Helmholtz-Platz, 76344 Eggenstein-Leopoldshafen, Germany; 3 Department of Physics, Federal University of Pelotas, PO Box 354, Pelotas, RS 96010-900 Brazil; 4 Department of Civil Engineering, Federal University of Amazonas, Manaus, AM Brazil; 5 Chemistry Department, Federal University of Paraná, Curitiba, 81531-980 Brazil; 6 University of Brasília, Institute of Physics, Brasília-DF, 70919-970 Brazil; 7 Department of Mathematics, Federal University of Amazonas, Manaus, AM Brazil; 8 Department of Physics, Federal University of Amazonas, Manaus, AM Brazil

**Keywords:** Energy science and technology, Engineering, Materials science, Physics

## Abstract

Here, we performed a systematic DFT study assisted by the workflow framework SimStack for the mechanical and thermodynamic properties of the clay mineral lizardite in pristine and six different types of O vacancies configurations. In most cases, the defect caused a structural phase transition in the lizardite from the trigonal (pristine) to the triclinic phase. The results show that oxygen vacancies in lizardite significantly reduce the lattice thermal conductivity, accompanied by an elastic moduli reduction and an anisotropy index increase. Through the *P–V* relation, an increase in compressibility was evidenced for vacancy configurations. Except for the vacancy with the same crystalline structure as pristine lizardite, the sound velocities of the other vacancy configurations produce a decrease in these velocities, and it is essential to highlight high values for the Grüneisen parameter. We emphasize the great relevance of the punctual-defects introduction, such as O vacancies, in lizardite, since this microstructural design is responsible for the decrease of the lattice thermal conductivity in comparison with the pristine system by decreasing the heat transfer ability, turning lizardite into a promising candidate for thermoelectric materials

## Introduction

Mineral clays are fascinating geological materials necessary in geophysics and potential technological applications. These materials possess plasticity when interacting with water and transform into ceramics in their absence, with prehistoric records of their applicability. Their structure comprises flat hexagonal sheets, and a serpentine family is a prominent group, with chrysotile, antigorite, and lizardite as the main members. They all have a similar chemical composition: Mg$_{3}$(Si$_{2}$O$_{5}$)(OH)${_4}$, composed of Si ion tetrahedrally coordinated with four O atoms and an Al or Mg ion octahedrally coordinated with six O or OH^1^, but different structures, depending on the arrangement of the silica-based tetrahedra to the aluminum- or magnesium-based octahedra^2^. Among these, lizardite (space group P31m and trigonal crystal structure^3,4^) is particularly noteworthy due to its abundance, stability under ambient conditions, and quasi-two-dimensional system, which favors its nanotechnology applications^5^. However, further studies are necessary to fully understand its mechanical properties, thermal transport, and thermodynamics in general from a compositional/constitutional understanding point of view.

Lizardite is the product of the metamorphic alteration of ultramafic igneous rocks that results in a crystalline structure that alternates silicate-tetrahedral (corner-shared SiO$_{4}$ units) and Mg-octahedral (edge-shared MgO${_2}$(OH)$_{4}$ octahedra) layers, forming a stacking of 1:1 sheets held together by weak hydrogen bonds. This intriguing structural arrangement, which is prone to volume swelling/shrinkage depending on changes in water content, needs further investigation from a theoretical and experimental point of view. As it is challenging to synthesize lizardite crystals of sufficient size to be used in experimental studies, there have been several theoretical studies based on semi-empirical methods^6^ and *ab initio* calculations based on density functional theory (DFT)^7–9^. Such studies focus on addressing structural, elastic, vibrational, and bonding properties, concentrating special attention on the pristine lizardite structure without considering its possible realistic constitution in nature, i.e., the defects in its structure.

From pristine lizardite studies, Auzende et al.^6^ have studied the elastic properties and the interaction energy between layers, as a function of pressure (*P*) and temperature *T*, of the hydrous phyllosilicate lizardite, using atomistic calculations and the third-order Birch-Murnaghan expression^10,11^, which is an appropriated approach to obtain the silicate^12–14^ and phyllosilicates^15–17^ crystalline structures and mechanical properties. In complete agreement with Auzende et al.^6^, Reynard et al.^7^ has obtained the elastic properties of lizardite via DFT at static conditions, where they have got the elastic stiffness constants and elastic compliances constants. On the other hand, Mookherjee and Stixrude^8^ performed *ab initio* calculations for lizardite under pressure, where they calculated the elastic stiffness constants and observed that the Birch-Murnaghan equation of state is not appropriate to describe the pressure-volume relationship over the entire range including low (from 0 to 7 GPA), intermediate (from 7 to 22 GPa), and high (greater than 22 GPa) pressure regions. Subsequently, the softening in the c-axis direction was associated with the abrupt decay at 10 GPa of the elastic stiffness constants^18^. Very recently, Deng et al.^9^ presented a comprehensive DFT-based study on the lizardite elastic properties at *P-T* conditions of subduction zones, showing that lizardite has significant shear wave anisotropy and arguing that its large elastic anisotropy could result from the shear-wave splitting in the subducting slabs.

Examining lizardite’s natural state is crucial to providing a detailed understanding resulting from myriad interactions and distinct chemical environments. The structural composition of lizardite is influenced by a range of defects, including the presence of trace elements such as (Mn, Fe, Co, Ni, Zn, and Al)^19^. Furthermore, the structure accommodates interstitial H$_{2}$O molecules^20–22^, and critically, vacancies. Recently, Gusmão et al.^5^ employed DFT calculations to scrutinize the impact of Mg cation substitution by Ca, Mn, Fe, Ni, and Zn on lizardite’s elastic properties. Their findings revealed noticeable shifts in the mechanical resistance and anisotropy of the resulting compounds compared to pure lizardite. Another noteworthy study by Sun et al.^23^ explored the enthalpy formation for H$_{2}$O defects during the dehydration process and found substantial alterations in lizardite’s elastic properties, affecting seismic velocities and their anisotropy. However, it’s important to underscore that there is a considerable gap in the existing literature when it comes to vacancy-type defects in lizardite. Despite the advancements in understanding the material’s behavior due to other kinds of defects, vacancy-type defects remain an unexplored avenue, which we try to unravel here.

Considering oxygen-abundance in lizardite composition, the most likely vacancy-type are by oxygen ones, which also happens in perovskite- (ABO$_{3}$)^24–28^ and scheelite-type (ABO$_{4}$, where A and B are cations)^29–31^ structures. Thus, the same behavior is expected for lizardite, i.e., the oxygen-vacancy occurrence, because of the crystal growth process, annealing, and/or substitutions inside the crystalline structure, significantly changing these materials’ electronic, optical, and mechanical properties. Therefore, we have performed a systematic study on the oxygen-vacancy effects on the lizardite (Mg$_{3}$(Si$_{2}$O$_{5}$)(OH)$_{4}$) related to their mechanical and thermodynamic properties through the first-principles calculations based on DFT assisted by the workflow framework SimStack, considering lizardite pristine and with oxygen vacancies, for six vacancy-types, aiming to decrease its heat transfer ability, which is associate with better thermoelectric efficiency. From our calculation, we observed that the oxygen vacancies reduce the lattice thermal conductivity of lizardite. Materials with low lattice thermal conductivity are essential for thermoelectric applications. The maximum efficiency in converting heat into electricity by a thermoelectric device is expressed by the figure of merit $ZT=\sigma S^2T/(\kappa _e+\kappa _L)$, where $\sigma$ is the electronic conductivity, *S* is the Seebeck coefficient, *T* is the temperature and $\kappa _e$ is the thermal electronic conductivity. Since $\sigma$, *S*, and $\kappa _e$ are interdependent and vary proportionally, an exciting strategy to enhance *ZT* is to search for materials with small lattice thermal conductivity $\kappa _L$. In this scenario, lizardite with oxygen vacancy can be a good candidate for application in thermoelectric technologies. Section “Methodology” presents the methodology with the atomic configurations, computational method, scientific workflow, and property analyses; Section “Results and discussion” shows the results and discussion; and in the last section, we have the conclusions.

## Methodology

### Atomic configurations

**Figure 1:**
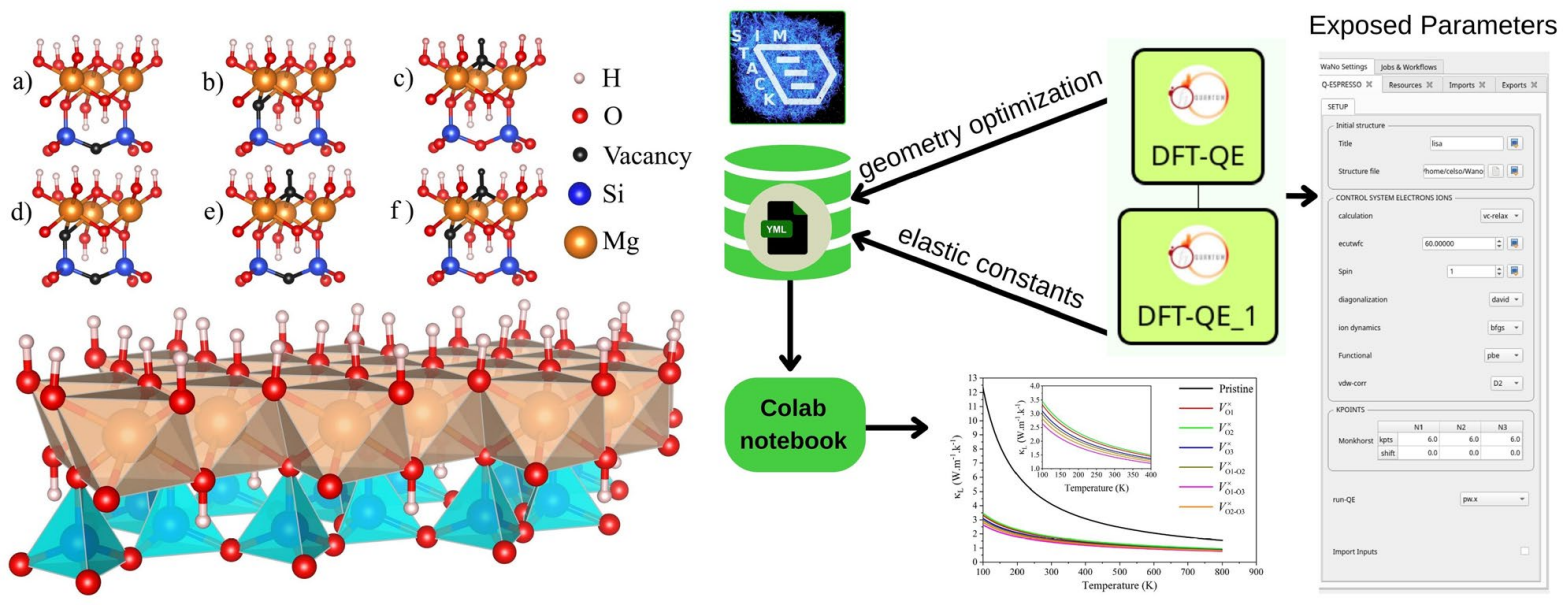
Left bottom: Mg$_{3}$(Si$_{2}$O$_{5}$)(OH)$_{4}$ one-layer representation, consisting of two sheets: an octahedra sheet, with Mg atom at the center of the octahedron; over a tetrahedra sheet, with Si atom at the center of the tetrahedron. Each Mg (Si) atom is surrounded by six (four) O atoms, and the atomic site on the octahedron top is occupied by OH. Top: the six types of oxygen vacancies: (**a**) removal of one (basal) O atom that bonds two Si atoms at the base of the tetrahedra sheet (V$^{\times }_{\text {O1}}$); (**b**) elimination of one (apical) O atom that bonds one Si atom in the tetrahedra sheet with three Mg atoms in the octahedra sheet (V$^{\times }_{\text {O2}}$); (**c**) elimination of one OH from the top of the octahedra sheet (V$^{\times }_{\text {O3}}$); (**d**) double vacancy originated from the simultaneous V$^{\times }_{\text {O1}}$ & V$^{\times }_{\text {O2}}$ vacancies (V$^{\times }_{\text {O1-O2}}$); (**e**) double vacancy originated from the simultaneous V$^{\times }_{\text {O1}}$ & V$^{\times }_{\text {O3}}$ vacancies (V$^{\times }_{\text {O1-O3}}$); and (**f**) double vacancy originated from the simultaneous V$^{\times }_{\text {O2}}$ & V$^{\times }_{\text {O3}}$ vacancies (V$^{\times }_{\text {O2-O3}}$). On the right side: We display the workflow that we used to do all the simulations and calculate the properties of Lizardite. In the steps DFT-QE and DFT-QE_1, we optimized geometry and the elastic constants. These steps created a database that we then fed into a Colab notebook to calculate lattice thermal conductivity ($\kappa _L$).

The Mg$_{3}$(Si$_{2}$O$_{5}$)(OH)$_{4}$ lizardite structure is a layered trigonal crystal with a space group P31m^3,4^ which has been studied in its pristine form and with six different types of oxygen vacancies, for which, we obtained the structural, mechanical, and thermodynamic properties. Following the Kroger-Vink notation^32^, we have organized the oxygen vacancies as follows: V$^{\times }_{\text {O1}}$ – formed by removing one (basal) O atom that bonds to two Si atoms in the tetrahedra layer; V$^{\times }_{\text {O2}}$ – by removing one (apical) O atom that bonds to a Si atom in the tetrahedra layer with three Mg atoms in the octahedra layer; V$^{\times }_{\text {O3}}$ – by removing one OH from the top of the octahedra layer; the double vacancies: V$^{\times }_{\text {O1-O2}}$, V$^{\times }_{\text {O1-O3}}$, and V$^{\times }_{\text {O3-O3}}$ are originated from the simultaneous formation of the vacancy pairs: V$^{\times }_{\text {O1}}$ & V$^{\times }_{\text {O2}}$, V$^{\times }_{\text {O1}}$ & V$^{\times }_{\text {O3}}$, and V$^{\times }_{\text {O2}}$ & V$^{\times }_{\text {O3}}$, respectively. For clarity, Fig. 1 can be consulted to differentiate the six types of oxygen vacancies (top) and for a more precise notion of the layered lizardite structure (bottom).

### Total energy calculations

Our *ab initio* calculations are based on DFT, as implemented in the Quantum ESPRESSO (QE) package^33^, using the ultrasoft method^34^ within the generalized gradient approximation with the semilocal Perdew–Burke–Ernzerhof (PBE)^35^ formulation for the exchange-correlation energy functional. As DFT-PBE cannot provide an accurate description of the nonlocal long-range van der Waals (vdW) interactions, we employ the vdW D2 correction proposed by Grimme^36,37^, which possess a good compromise between accuracy and computational coast^38,39^. The geometry optimization was carried out by the Broyden–Fletcher–Goldfarb–Shanno (BFGS) quasi-Newton algorithm^35^, with the convergence thresholds on forces and total energy for ionic minimization established in $10^{-6}$ eV/Å and $10^{-5}$ eV, respectively. The kinetic energy cutoffs related to the wave functions and charge density calculations in the summation were 0.8 keV and 8.2 keV, respectively. After convergence tests, a Monkhorst-Pack scheme was used for the Brillouin zone integration, with a $6\times 6\times 6$ ${\textbf {k}}$-point mesh. The graphical representations of the structural models were performed using the VESTA package^40^.

### SimStack workflow

SimStack^41^ is a robust workflow framework that ensures the reproducibility and transferability of the simulation protocols^42^. Additionally, it simplifies the creation of custom-tailored simulation protocols using various computer simulation approaches. Here we use the Workflow Active Node (WaNo) DFT-QE^43^, which saves time by automating and reducing protocol complexity, permitting the monitoring of multiple sets of calculations for independent DFT protocols for different Lizardite configurations. The right side of Fig. 1 depicts this workflow, streamlining the optimization and elastic constants calculations using QE code. The output of DFT simulations for each Lizardite system automatically generates *.yml* file database, which we automatically may transfer to a Colab notebook to query data and calculate the lattice thermal conductivity $\kappa _L$. To meet the FAIR principles^44^, we make all input data and the Colab notebook available on the following repository github.com/KIT-Workflows/Lizardite.

### Property analyses

To deepen the understanding of lizardite, we have systematically analyzed its main mechanical and thermodynamic properties, including the lattice thermal conductivity. Further details on the properties are provided in the Supporting Information (SI) material. In terms of mechanical properties, we had considered the elastic stiffness ($c_{ij}$) and compliance ($s_{ij}$) constants (and their relations) as implemented in the Thermo_pw package^45^, checking the stability criterion when the eigenvalues of the elastic stiffness matrix were greater than zero^46–49^. Posteriorly, assuming the relaxation process under hydrostatic pressure^50^, the Voigt and Reuss bulk ($B_V$, $B_R$) and shear ($G_V$, $G_R$) moduli, respectively, were obtained from $c_{ij}$ and $s_{ij}$. From which, the Hill bulk ($B_H$) and shear ($G_H$) moduli are given by averaging the corresponding values^50^: $B(G)_H = 1/2(B(G)_V+B(G)_R)$. Considering Hill’s modulus and the mathematical theory of elasticity^51^, the Young’s modulus ($E_H$) and Poisson’s ratio ($\nu _H$) were also considered. At the same time, the anisotropy of the material was obtained from the universal elastic anisotropy index ($A^U$)^52^. Finally, we also have the possibility of estimating the ductility or brittleness of material through the criterion suggested by Pugh^53^, for which ratios $B_H/G_H$ larger than 1.75 represent a ductile material.

Considering a *P–V* equations of state, the thermodynamic properties were obtained from the structural relaxation process under a pressure range from 0.0 to 8.0 GPa. The isochoric heat capacity ($C_V$) was achieved by post-processing with Thermo_pw package^45^, considering the Debye temperature ($\Theta _D$) within the Debye model. To get $\Theta _D$, the elastic constant data was considered through the Voigt–Reuss–Hill average of the bulk and shear moduli to calculate the average sound velocities. Following Anderson^54^, where $\Theta _D$ depends on the average sound velocity ($v_{av}$), which is given in terms of the longitudinal ($v_\ell$) and transverse ($v_t$) sound velocities, i.e., $v_{av}=[1/3(1/v_{\ell }^3+2/v_{t}^3)]^{-1/3}$. In turn, $v_\ell$ and $v_t$ depends on the mechanical properties ($B_H$ and $G_H$)^51,55^. Similarly, the Grüneisen acoustic constant ($\gamma$) was calculated as a $v_\ell$ and $v_t$ function, following Belomestnykh^56^. See more details about thermodynamic properties in SI.

For the lattice thermal conductivity ($\kappa _L$), we have considered two models: (*i*) for the pristine lizardite, the Slack model^57,58^ has been adopted since it is widely appropriated for defect-free crystals and lattice thermal resistance resulting only from intrinsic phonon-phonon interactions^59–62^. The $\kappa _L$ is obtained from $\Theta _D$ and $\gamma$, which can be obtained from lattice dynamic calculations or experimental measurements^57,61^. However, in the present work, we have obtained $\kappa _L$ considering directly the $\Theta _D$ and $\gamma$ calculation (see SI for mathematical expressions), in complete agreement with Xia et al.^63^ approach. (*ii*) For lizardite with oxygen vacancies, $\kappa _L$ is calculated following the seminal works of Klemens^64,65^ and corroborated by Callaway et al.^66^ and Abeles^67^. Basically, $\kappa _L$ for lizardite with an oxygen vacancy type ($V^{\times }_{\alpha }$) is defined as $\kappa _{V^{\times }_{\alpha }}=\kappa _L[(\tan ^{-1}u)/u]$, i.e., it is given in terms of $\kappa _L$ for the pristine lizardite and *u* is written as a function of $\Theta _D$, $\kappa _L$, $v_{av}$, and $\Gamma$ (which is given in terms of the concentration and mass of the atom type *i*). Finally, the minimum lattice thermal conductivity of lizardite with oxygen vacancy ($\kappa _{V^{\times }_{\alpha }(\text {min})}$) is obtained using the minimum value of $\kappa _L$ for pristine lizardite ($\kappa _{L(\text {min})}$), according to Clarke^68^. More details are presented in the SI material.

In Table 1 we present the accuracy of the Belomestnykh approximation used in the present work to determine the Grüneisen parameters, in relation to the DFT calculation , for some examples from literature for structure simpler than the lizardite one. It is observed that the results from the Belomestnykh approach are in reasonable agreement with the DFT calculations.

**Table 1:** Comparision of the Grüneisen parameters obtained from the Belomestnykh approximation with the DFT calculation for some compounds.

Compound	$\gamma$ (DFT)	$\gamma$(Belomestnykh)
Mg$_{3}$Sb$_{2}$	1.83^(a)^	1.85^(a)^
CaMg$_{2}$Sb$_{2}$	1.44^(a)^	1.40^(a)^
CaMg$_{2}$Bi$_{2}$	1.46^(a)^	1.45^(a)^
Bi$_{2}$Te$_{3}$	1.52^(b)^	1.65^(c)^
SnSe	2.83^(d)^	3.13^(d)^
PbTe	1.49^(d)^	1.65^(d)^
PbSe	2.66^(d)^	1.69^(d)^
PbS	2.46^(d)^	1.67^(d)^

(a) Ref.^69^

(b) Ref.^70^

(c) Ref.^71^

(d) Ref.^63^

## Results and discussion

### Geometric optimization

We have performed the structural optimization of pristine and oxygen vacancies lizardite within the stacking layer context, each of which is composed of two sheets: a Mg-centered octahedra over a Si-centered tetrahedra sheet, as shown in the bottom of Fig. 1. The six types of O-vacancies considered are given by: V$^{\times }_{\text {O1}}$, V$^{\times }_{\text {O2}}$, V$^{\times }_{\text {O3}}$, V$^{\times }_{\text {O1-O2}}$, V$^{\times }_{\text {O1-O3}}$, and V$^{\times }_{\text {O2-O3}}$, as shown at the top of Fig. 1 (from a) to f), respectively). The structural relaxation procedure of these systems and the subsequent comparison between the pristine and defect systems are of great relevance for establishing the main structural changes resulting from vacancy formation. Thus, the main structural properties discussed here, e.g., lattice parameters (*a*, *b*, *c*), angles ($\alpha$, $\beta$, $\gamma$), volume, density, and interlayer distance, are also presented in SI (Table S1).

For the pristine lizardite, we have obtained a trigonal lattice with parameters: $a = b = 5.276~\text{\AA }$ and $c = 7.117~\text{\AA }$; $\alpha = \beta = 90\mathrm {{}^\circ }$ and $\gamma = 120\mathrm {{}^\circ }$, and a layer distance of $1.783~\text{\AA }$, results that are in excellent agreement with previous works^5^. The formation of different types of vacancies in lizardite can lead to further structural parameter responses. In this context, we initially estimated the formation viability of the O-vacancies studied based on the formation energy ($\Delta E_\text {v}^\text {o}$) as established by Emery and Wolverton^72^:

1 $$\begin{aligned} \Delta E_\text {v}^\text {o}=E({Mg_{3}Si_{2}O_{(5-\alpha )}(OH)_{(4-\beta )}})+\alpha \mu _\text {O} +\beta \mu _\text {H}-E({Mg_{3}Si_{2}O_{5}(OH)_{4}}), \end{aligned}$$

where $E({Mg_{3}Si_{2}O_{(5-\alpha )}(OH)_{(4-\beta )}})$ is the total energy of Mg$_{3}$Si$_{2}$O$_{5}$(OH)$_{4}$ with vacancy, $\mu _\text {O}$ and $\mu _\text {H}$ are the O and H chemical potentials, respectively, $E({Mg_{3}Si_{2}O_{5}(OH)_{4}})$ is the total energy of the pristine Mg$_{3}$Si$_{2}$O$_{5}$(OH)$_{4}$, and $\alpha$ ($\beta$) is the number of O atoms removed from a tetrahedron vertex (the top of the octahedra sheet), and zero otherwise. The $\Delta E_\text {v}^\text {o}$ values, along with the percentage differences in the volume ($\Delta Vol$) and interlayer distance ($\Delta id$) concerning the pristine system, are shown in Table 2.

**Table 2:** The formation energy of O vacancy per oxygen atom ($\Delta E_\text {v}^\text {o}$), the percentage difference in the volume of structures concerning the pristine one ($\Delta Vol$), and the percentage difference in the interlayer distance ($\Delta id$) for the pristine system, for different types of vacancies in lizardite. Negative (positive) $\Delta Vol$ and $\Delta id$ values represent a decrease (increase) in the volume and interlayer distance of the structures with pristine lizardite.

Vacancy	V$^{\times }_{\text {O1}}$	V$^{\times }_{\text {O2}}$	V$^{\times }_{\text {O3}}$	V$^{\times }_{\text {O1-O2}}$	V$^{\times }_{\text {O1-O3}}$	V$^{\times }_{\text {O2-O3}}$
$\Delta E_\text {v}^\text {o}$ (eV)	0.824	0.773	0.874	1.592	1.665	1.665
$\Delta Vol$ (%)	− 4.83	− 2.67	0.39	− 6.45	− 5.20	− 2.75
$\Delta id$ (%)	− 2.36	− 5.89	− 0.17	− 9.48	− 7.12	− 8.02

From Table 2, it is observed that the lowest formation energy is obtained for V$^{\times }_{\text {O2}}$ vacancy-type, followed by V$^{\times }_{\text {O1}}$ and V$^{\times }_{\text {O3}}$. In contrast, the formation energy of a double vacancy is approximately the sum of the energies of the two single vacancies that originated it. This result is directly associated with the fact that only V$^{\times }_{\text {O2}}$ preserved the trigonal lattice structure in agreement with the pristine lizardite. At the same time, the other vacancy systems changed the lattice structure type from trigonal to triclinic. For V$^{\times }_{\text {O2}}$, the removal of an oxygen atom between the octahedra and tetrahedra sheets causes a moderate volume contraction ($\Delta Vol = -$2.67%), which is directly linked to an approximation between the sheets ($\Delta id = -$5.89%) concerning pristine lizardite, all without changing the lattice angles of the trigonal structure. On the other hand, all different vacancy types represent structural changes in the pristine lizardite that, to a greater or lesser extent, lead to distortions that penalize the stabilization of the system with point defects, with volume variations ranging from a slight expansion ($\Delta Vol =$ 0.39%) for V$^{\times }_{\text {O3}}$, due to the small approximation between the sheets ($\Delta id = -$0.17%), to significant volume contractions ($\Delta Vol = -$6.45%) for V$^{\times }_{\text {O1-O2}}$, due to the largest approximation between sheets ($\Delta id = -$9.48%), all of them within the structural context of the triclinic lattice. In short, the six vacancy types studied here represent, together with pristine lizardite, a very diversified set from the point of view of possible structural alterations. At the same time, they are energetically viable possibilities to happen in a practical application.

### Mechanical properties

We started the study of the mechanical properties of pristine and vacancy-type lizardite from the calculation of the elastic stiffness constants $c_{ij}$ and the elastic compliances constants $s_{ij}$, which are presented in the SI (Table S2). From this, we performed a two-level characterization of the main mechanical properties, a primary and complementary description of the mechanical response properties of the studied materials. Firstly, from the matrices [$c_{ij}$] and [$s_{ij}$], it is possible to check the mechanical stability criterion, which establishes that all eigenvalues of these matrices must be greater than zero to achieve the elastic stability^46–49^. All structures studied here meet this criterion, showing elastic stability in pristine and vacancy-type constitutions. Our results are in excellent agreement with the literature for the case of pristine lizardite^8^.

Within the context of basic mechanical properties, we can check on (*i*) the in-plane and out-of-plane mechanical stiffness, (*ii*) the fracture resistance (or rigidity), and (*iii*) the stress anisotropy of the lizardite configurations with and without defect, which turns out to be interesting in the scenario of how pristine and vacancy-types lizardite resists concerning the stress application. For (*i*), an estimate can be made through the ratio $c_{11}/c_{33}$, whose values are presented in Table 3. We obtain a ratio $c_{11}/c_{33} = 1.707$ for the pristine configuration, which implies a greater mechanical stress resistance in-plane than out-of-plane. As expected, for configurations with vacancy, we have a stiffness dependence on the sites from which the O atom has been removed. Except for V$^{\times }_{\text {O1-O3}}$, for which the in-plane and out-of-plane stiffness are equivalent, all other cases of vacancy cases behave like the pristine lizardite, i.e., are weaker bonds along the [001] direction. For (*ii*), the estimate is made by the ratio $c_{66}/c_{44}$, which represents how much the basal plane is more resistant to fracture than the axis perpendicular to this plane when shear stress is applied. The rigidity values ($c_{66}/c_{44}$) are presented in Table 3. We observed that, with pristine lizardite, the basal plane is more (less) resistant to fractures than the c-axis for V$^{\times }_{\text {O1}}$, V$^{\times }_{\text {O3}}$, V$^{\times }_{\text {O1-O3}}$ and V$^{\times }_{\text {O2-O3}}$ (V$^{\times }_{\text {O2}}$ and V$^{\times }_{\text {O1-O2}}$). Finally, for (*iii*), we can estimate the stress anisotropy from the linear compressibility ($\beta$), which represents the relative variation in the length of a line when the body is subjected to unit hydrostatic pressure^73^ and depends on the elastic compliance constants $s_{ij}$. Following the Nye work^49^, we have estimated $\beta$ for trigonal and triclinic crystal systems in each axial direction, as follows: $\beta _1=s_{11}+s_{12}+s_{13}$ (both), $\beta _2=\beta _1$ (trigonal), $\beta _2=s_{12}+s_{22}+s_{23}$ (triclinic), $\beta _3=2s_{13}+s_{33}$ (trigonal), and $\beta _3=s_{13}+s_{23}+s_{33}$ (triclinic). The $\beta$ values are also presented in Table 3, in which we observe that the two configurations with trigonal lattice (pristine and V$^{\times }_{\text {O2}}$) present isotropic in-plane stiffness ($\beta _1=\beta _2$) as expected, due to the crystalline symmetry. In contrast, the other vacancy-types lizardite configurations show planar anisotropy.

**Table 3:** The in-plane and out-of-plane mechanical stiffness ($c_{11}/c_{33}$), fracture resistance ($c_{66}/c_{44}$), and linear compressibilities ($\beta _1$, $\beta _2$, and $\beta _3$) of the lizardite with and without O vacancies. The linear compressibilities are given in 1/Mbar=1/100GPa.

Compound	$c_{11}/c_{33}$	$c_{66}/c_{44}$	$\beta _1$	$\beta _2$	$\beta _3$
Pristine	1.707	3.630	0.247	0.247	0.607
V$^{\times }_{\text {O1}}$	1.650	6.120	0.349	0.306	0.792
V$^{\times }_{\text {O2}}$	1.440	3.380	0.275	0.275	0.541
V$^{\times }_{\text {O3}}$	2.050	7.370	0.324	0.351	1.061
V$^{\times }_{\text {O1-O2}}$	1.190	3.570	0.458	0.347	0.652
V$^{\times }_{\text {O1-O3}}$	0.990	3.730	0.687	0.489	0.957
V$^{\times }_{\text {O2-O3}}$	1.740	4.170	0.367	0.310	0.891

Complementarily, using the elastic constants tensor and the Voigt, Reuss, and Hill models, we have obtained the bulk ($B_V$, $B_R$), shear ($G_V$, $G_R$), and Young ($E_H$) moduli, as well as the Poisson’s ratio ($\nu _H$) and the universal elastic anisotropy index ($A^U$) for a complete mechanical property characterization of the pristine lizardite and with different O vacancy-types. All these properties are shown in Table 4. First, we observe that the vacancy formation leads to a decrease in the $B_V$, $B_R$, $G_V$, $G_R$, and $E_H$ values, which is directly associated with the degree of the constitutional importance of the O atom(s) removed to form the vacancy. For example, the smallest elastic modulus values occur for a double vacancy, specifically for V$^{\times }_{\text {O1-O3}}$, when two O atoms are removed, one bonded to two Si atoms at the base of the tetrahedra sheet and the other at the top of the octahedra sheet. Even more specifically, the V$^{\times }_{\text {O3}}$ vacancy type leads to elastic moduli smaller than V$^{\times }_{\text {O1}}$ one, i.e., removing a O atom from the top of the octahedra sheet decreases the elastic moduli more than removing a O atom from the base of the tetrahedra sheet. All studied configurations prefer to be shear deformed since the shear moduli are smaller than the bulk moduli. In the case of the $\nu _H$ values, we observed a slight variation ($\nu _H = 0.30 \pm 0.01$). Similarly to the small variation of the ratio $B_H/G_H$, i.e., between 2.01 and 2.24, which, according to the criterion suggested by Pugh^53^, characterizes all our configurations as ductile, since the values are more significant than 1.75. Finally, the smallest $A^U$ values occur for the trigonal structures (pristine and V$^{\times }_{\text {O2}}$), which are isotropic in-plane in terms of stiffness, in contrast to triclinic (all other cases), in complete agreement with the linear compressibility results.

**Table 4:** The bulk ($B_V$, $B_R$), shear ($G_V$, $G_R$), and Young ($E_H$) moduli (in GPa); the Poisson’s ratio ($\nu _H$) and the universal elastic anisotropy index ($A^U$) of the lizardite with and without vacancies, where the subscript-labels: *V*, *R*, and *H* refer to the Voigt, Reuss, and Hill models, respectively. In parentheses, we have added the respective property values of the pristine lizardite from literature^8^.

Compound	$B_V$	$G_V$	$B_R$	$G_R$	$E_H$	$\nu _H$	A${}^{U}$
Pristine	101.84	53.45	90.85	35.74	115.64	0.30	2.60
	(95.66)	(53.85)	(80.70)	(36.23)	(115.46)	(0.28)	(2.63)
V$^{\times }_{\text {O1}}$	79.71	47.89	69.06	23.96	92.06	0.29	5.15
V$^{\times }_{\text {O2}}$	97.57	50.81	91.59	35.73	112.37	0.30	2.18
V$^{\times }_{\text {O3}}$	70.85	39.76	57.59	19.88	76.99	0.30	5.23
V$^{\times }_{\text {O1-O2}}$	73.02	42.76	68.63	27.75	90.34	0.29	2.77
V$^{\times }_{\text {O1-O3}}$	57.44	31.59	46.87	15.08	60.47	0.31	5.70
V$^{\times }_{\text {O2-O3}}$	74.78	40.11	63.81	23.26	82.15	0.30	3.80

### Thermodynamic properties

**Figure 2:**
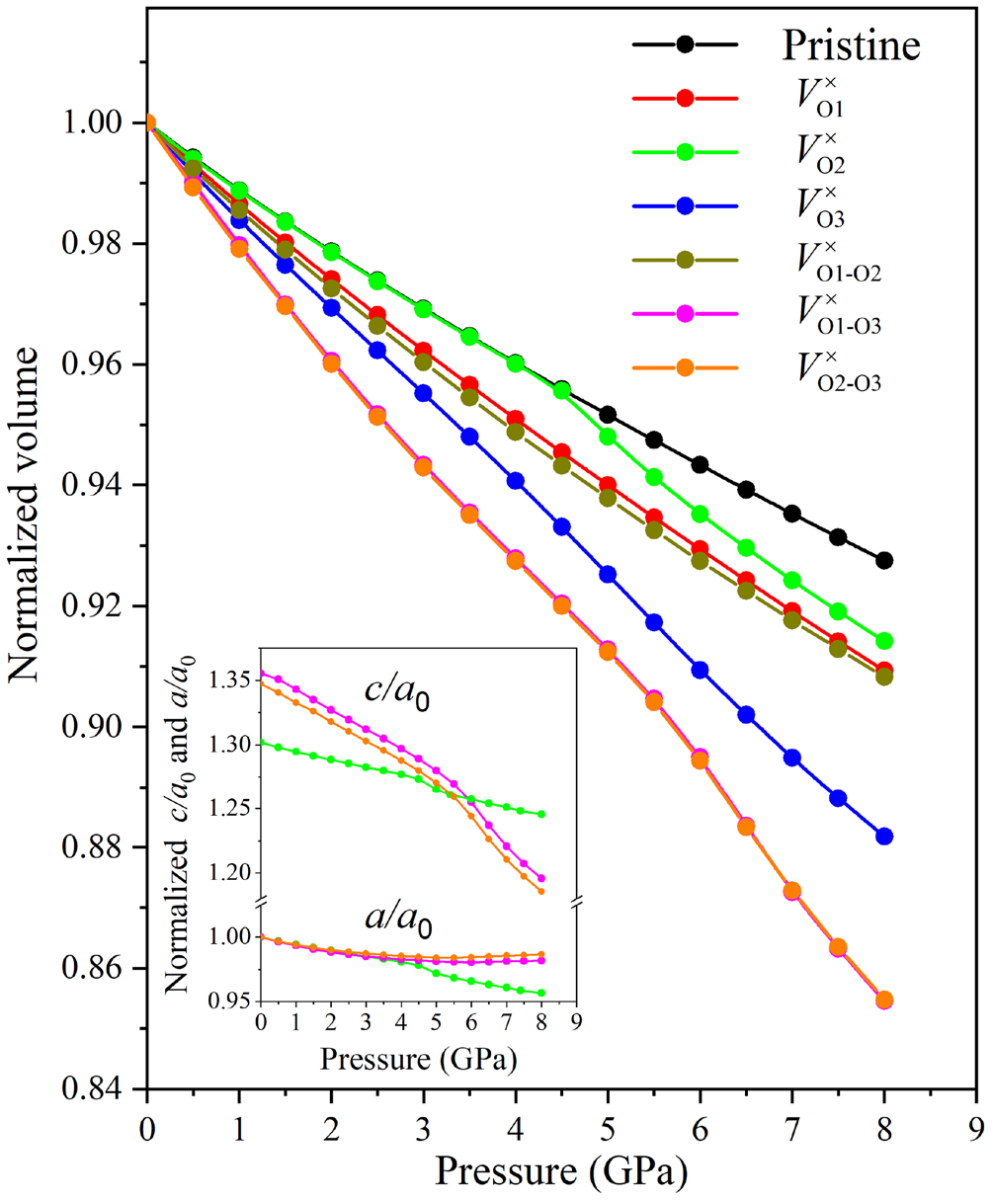
Variation of normalized volume in relation to pressure for pristine and O vacancy-types lizardite. Two inserts are shown for the normalized (scaled by $a_0$, which consists of *a* at zero pressure) lattice parameters (*a* and *c*) variation in relation to pressure to explain the escape from a linear behavior for the *P–V* diagrams of the V$^{\times }_{\text {O2}}$, V$^{\times }_{\text {O1-O3}}$, and V$^{\times }_{\text {O2-O3}}$ configurations.

Once the mechanical properties of pristine and vacancy-types lizardite were established, there is a need for a thermodynamic property analysis taking into account pressure and temperature variations to trace the real implications of O vacancies in the lizardite constitution. First, in terms of the pressure variation, we obtained the *P–V* equations of state from the structural relaxation under pressure (from zero to 8.0 GPa), which are presented in Fig. 2. The normalized volume (relative to the structure at zero pressure) decreases with increasing pressure for all configurations, and the *P–V* curve shows a greater slope with O vacancies, i.e., the lizardite configurations with vacancies present a more significant decrease in volume with increasing pressure, which is directly correlated with the mechanical properties elucidated by the bulk, shear, and Young modulus values (see Table 4). Up to 4.5 GPa, the *P–V* curves of the pristine and V$^{\times }_{\text {O2}}$ configurations have the same response to pressure increase, keeping in mind that both have the trigonal crystalline structure; for values larger than 4.5 GPa, we observe that the V$^{\times }_{\text {O2}}$ configuration starts to have a more accentuated response to the pressure increase, which is associated with the structural change that is evident from the lattice parameters (*a* and *c*) variation in the insert of Fig. 2. All other configurations with vacancy, possessing the triclinic crystalline structure, are more susceptible to pressure variations, respond more accentuated, and are more compressible. The most significant decrease in volume with the pressure increase occurs for V$^{\times }_{\text {O1-O3}}$ and V$^{\times }_{\text {O2-O3}}$ (whose *P–V* diagrams are practically overlapping), which are the two configurations with double vacancies that have the highest (and identical) vacancy formation energies. These three lizardite configurations (V$^{\times }_{\text {O2}}$, V$^{\times }_{\text {O1-O3}}$, and V$^{\times }_{\text {O2-O3}}$) had a change in the expected linear behavior between volume and pressure, showing an increase in the slope from 4.5 GPa, which is associated with the rise in compressibility that occurs mainly on the *c* axis, which implies a reduction in the interlayer space and significant changes in the O–H–Mg angle, positioning O–H outside the normal direction.

For the temperature variation, we performed the isochoric heat capacity ($C_V$) calculation, which first requires obtaining $\Theta _D$ from the Debye model, whose expression depends on $v_{av}$, given by the angular average of the sound velocities calculated for each propagation direction, i.e., $v_\ell$ and $v_t$, both being important for the Grüneisen acoustic constant ($\gamma$) determination. Thus, before we get into $C_V$, we briefly discuss the $v_\ell$, $v_t$, $v_{av}$, $\gamma$, and $\Theta _D$ results for lizardite configurations with and without vacancies, as presented in Table 5. Our sound velocity results are in excellent agreement with the experimental results obtained for pristine lizardite. For example, Kern et al.^74^ have obtained $v_\ell$ and $v_t$ in the serpentine, at low pressure, around 7.2 and 4.8 km/s, respectively. According to Christensen^75^, which presented $v_\ell$ values in the range of 5.0–8.3 km/s, depending on the sample serpentinization. Or the work by Seipold and Schilling^21^, for which $v_{av}$ varies from approximately 5.02 km/s under ambient conditions to 4.5 km/s at 900 K.

**Table 5:** Longitudinal ($v_\ell$), transverse ($v_t$), and average ($v_{av}$) sound velocities; the ratio velocities ($v_\ell /v_t$); Grüneisen parameter ($\gamma$); the Debye temperature ($\theta _D$); and te the minimum thermal conductivity ($\kappa _{\text {min}}$), of the pristine and O vacancy-types lizardite.

Compound	$v_{\ell }$	$v_t$	$v_{av}$	$v_\ell /v_t$	$\gamma$	$\theta _D$	$\kappa _{\text {min}}$
	(km/s)	(km/s)	(km/s)			(K)	(W/(mK))
Pristine	7.62	4.08	4.55	1.87	1.77	639.60	1.75
V$^{\times }_{\text {O1}}$	6.79	3.68	4.10	1.84	1.72	574.91	0.63
V$^{\times }_{\text {O2}}$	7.66	3.08	4.56	1.88	1.78	634.09	0.67
V$^{\times }_{\text {O3}}$	6.44	3.45	3.85	1.87	1.77	519.40	0.55
V$^{\times }_{\text {O1-O2}}$	6.82	3.73	4.16	1.83	1.69	574.00	0.51
V$^{\times }_{\text {O1-O3}}$	5.78	3.06	3.42	1.89	1.81	460.11	0.40
V$^{\times }_{\text {O2-O3}}$	6.78	3.61	4.04	1.88	1.78	538.19	0.46

From Table 5, we observe that, in general, the different vacancy-types formation is associated with a sound velocity decrease, except for the pristine and V$^{\times }_{\text {O2}}$ configurations, the only vacancy configuration that preserves the trigonal crystalline structure. Unanimously, we found that $v_\ell$ is a bit more than 1.8 times $v_t$, which means that sound easily propagates in the plane of the layers than along the direction of the stacking layers ([001]). Our $v_\ell /v_t$ results agree with the experimental results of Christensen^75^, by which this ratio increases systematically from 1.78 to 2.21 with increasing sample serpentinization. We correlate the mechanical properties (Table 4) and the sound velocities. For example, V$^{\times }_{\text {O1-O3}}$ has the lowest sound velocities in correspondence to the smallest volume, shear modulus, and Young’s modulus. For $\gamma$, we observed a slight variation among configurations, leading to an average value of 1.76. On the other hand, $\Theta _D$ presents smaller values for the structures with vacancy, being the smallest values for the configurations with a triclinic crystalline lattice than with a trigonal one.

**Figure 3:**
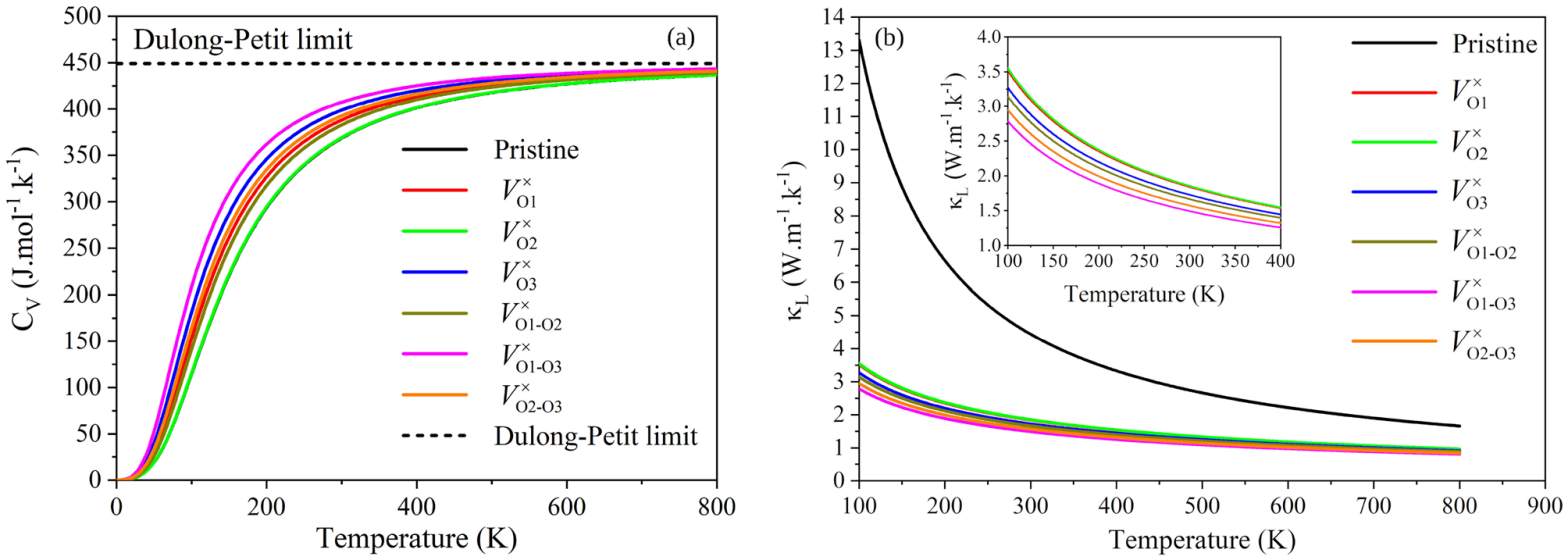
(**a**) The isochoric heat capacity ($C_V$) of pristine and O vacancy-types lizardite as a function of temperature. (**b**) The lattice thermal conductivity ($\kappa _L$) as a temperature function of pristine and O vacancy-types lizardite.

After these preliminary calculations, we performed the $C_V$ calculations concerning the temperature, from 0 to 800 K, for the lizardite configurations with and without O vacancies, which are presented in Fig. 3a. We observed the same behavior trend in the $C_V$ *versus* temperature curves for all lizardite configurations, with an exponential increase in $C_V$ up to approximately room temperature and subsequent logarithmic growth for $C_V$ at higher temperatures, with a trend asymptotic to converge to the Dulong-Petit limit at high temperatures. The most significant $C_V$ differences among the configurations occur in the 100–300 K interval, where we observe that, with the V$^{\times }_{\text {O2}}$ exception, the $C_V$ values are more prominent for the lizardite configurations with vacancy than that for pristine lizardite.

#### Lattice thermal conductivity

From $\gamma$ and $\Theta _D$, we can calculate the lattice thermal conductivity ($\kappa _L$), as well as the minimum thermal conductivity ($\kappa _{\text {min}}$)), which are shown in Table 5, for the pristine and O vacancy-types lizardite. The behavior trend between $\kappa _L$ and $\kappa _{\text {min}}$ is the same across the configurations studied, i.e., for $\kappa _{\text {min}}$ (in W/(m.K)): pristine (1.75) > V$^{\times }_{\text {O2}}$ (0.67) > V$^{\times }_{\text {O1}}$ (0.63) > V$^{\times }_{\text {O3}}$ (0.55) > V$^{\times }_{\text {O1-O2}}$ (0.51) > V$^{\times }_{\text {O2-O3}}$ (0.46) > V$^{\times }_{\text {O1-O3}}$ (0.40), in agreement with $\kappa _L$ as a temperature function, in Fig. 3b. As evidenced, the O vacancies formation in lizardite leads to a decrease in heat transfer ability compared to the pristine configuration.

Thus, from Fig. 3b, we observe that the O vacancies produce a significant $\kappa _L$ decrease, following the sound velocities (Table 5), which represents our main result here, since the $\kappa _L$ values, the material conducts less heat energy. Furthermore, we highlight that this important thermoelectric parameter, directly linked to determining the energy conversion efficiency of thermoelectric materials, has a very low variation with increasing temperature for systems with the vacancy in contrast to the pristine system. Consequently, the introduction of the defect (O vacancies) in lizardite is part of the successful strategy of intelligent microstructural design, which plays an essential role in the task of obtaining materials with high phonon-phonon scattering rates, i.e., materials with significant Grüneisen parameters. These behavior are in agreement with the results obtained by Shen et al.^76^ and Jia et al.^77^, which studied the effect of vacancies on the lattice thermal conductivity of CuGaTe$_{2}$ and In$_{2}$Te$_{3}$-InSb, respectively.

## Conclusions

In the present work, we performed a systematic first-principles study, based on DFT, for the main mechanical and thermodynamic properties of the clay mineral lizardite in pristine and six different types of O vacancies configurations. After the structural relaxation process, except for a vacancy configuration, we obtained a change in the crystalline structure of the vacancy configurations, going from trigonal (pristine) to triclinic. As the main result found, we highlight that oxygen vacancies in lizardite significantly reduce the lattice thermal conductivity, accompanied by an elastic moduli reduction and an anisotropy index increase. Through the *P–V* relation, an increase in compressibility was evidenced for vacancy configurations. Except for the vacancy with the same crystalline structure as pristine lizardite, the sound velocities of the other vacancy configurations produce a decrease in these velocities, and it is essential to highlight high values for the Grüneisen parameter. Finally, we emphasize the great relevance of the punctual-defects introduction, such as O vacancies, in lizardite, since this intelligent microstructural design procedure is responsible for the decrease of the lattice thermal conductivity in comparison with the pristine system, which decreases the heat transfer ability, giving rise to an essential candidate for thermoelectric materials.

### Supplementary Information

Supplementary Information 1.

Supplementary Information 2.

Supplementary Information 3.

Supplementary Information 4.

## Data Availability

The data sets used and/or analyzed during the current study are available on the following repository github.com/KIT-Workflows/Lizardite.
