# Supplementary material for: Unveiling oxygen vacancy impact on lizardite thermo and mechanical properties
Source: Sci Rep. 2023 Oct 11;13:17157. doi: 10.1038/s41598-023-44424-9 (PMC10567844; doi:10.1038/s41598-023-44424-9)
Supplement: Supplementary file 4 — Supplementary Information 4. [file 41598_2023_44424_MOESM4_ESM.pdf]

Supporting Information: Unveiling Oxygen Vacancy Impact on Lizardite Thermo and Mechanical Properties

H. Pecinatto,† Celso R. C. Rêgo,*,‡ W. Wenzel,‡ C. A. Frota,¶ B. M. S. Perrone,† Maurício J. Piotrowski,§ Diego Guedes-Sobrinho,‖ Alexandre C. Dias,⊥ Cicero Mota,# M. S. S. Gusmão,@ and H. O. Frota@

†PPG-FIS, Federal University of Amazonas, Manaus-AM, Brazil

‡Karlsruhe Institute of Technology (KIT), Institute of Nanotechnology Hermann-von-Helmholtz-Platz, 76344, Eggenstein-Leopoldshafen, Germany

¶Department of Civil Engineering, Federal University of Amazonas, Manaus-AM, Brazil

§Department of Physics, Federal University of Pelotas, PO Box 354, 96010-900, Pelotas, RS, Brazil

‖Chemistry Department, Federal University of Paraná, 81531-980, Curitiba, Brazil

⊥Instituto de Física, Universidade de Brasília, Brasília-DF 70919-970, Brazil

#Department of Mathematics, Federal University of Amazonas, Manaus-AM, Brazil

@Department of Physics, Federal University of Amazonas, Manaus-AM, Brazil

E-mail: celso.rego@kit.edu

Contents

1 Property analyses **S2**

1.1 Mechanical properties S2

1.2 Thermodynamic properties S3

1.2.1 Lattice thermal conductivity S4

2 Results **S7**

2.1 Structural parameters S7

2.2 Mechanical properties S7

2.3 Lattice thermal conductivity S11

3 Atomic positions in crystal coordinates **S12**

References **S15**

1 Property analyses

1.1 Mechanical properties

The elastic stiffness constants c_{ij} (in Voigt notation $c_{ijk\ell} \to c_{\alpha\beta}$) and the elastic compliance constants s_{ij} (in Voigt notation $s_{ijk\ell} \to s_{\alpha\beta}$) were determined according to the Thermo_pw package[1], a Quantum ESPRESSO (QE) driver, from the second-order elastic constant matrix c_{ij}=$(\partial^2 E/\partial\varepsilon_i\partial\varepsilon_j)/V_0$, where E is the crystal energy, ε is the strain, and V_0 is the equilibrium volume. Stability was verified following the criterion that the eigenvalues of the elastic stiffness matrix must all be greater than zero[2–5]. Assuming that the relaxation process occurs under hydrostatic pressure[6], the Voigt (Reuss) bulk B_V (B_R) and shear G_V (G_R) moduli were obtained from c_{ij} and s_{ij} as follows:

$$B_V = \frac{1}{9}[c_{11}+c_{22}+c_{33}+2(c_{12}+c_{13}+c_{23})] \quad (1)$$

$$G_V = \frac{1}{15}[c_{11}+c_{22}+c_{33}-(c_{12}+c_{13}+c_{23}) +3(c_{44}+c_{55}+c_{66})] \quad (2)$$

$$B_R = [s_{11}+s_{22}+s_{33}+2(s_{12}+s_{13}+s_{23})]^{-1} \quad (3)$$

$$G_R = 15[4(s_{11}+s_{22}+s_{33})-4(s_{12}+s_{13}+s_{23}) +3(s_{44}+s_{55}+s_{66})]^{-1}. \quad (4)$$

and, from the mathematical theory of elasticity[7], the Voigt (Reuss) Young moduli E_V (E_R) are obtained from

$$E_V = \frac{9B_V G_V}{3B_V + G_V} \quad (5)$$

$$E_R = \frac{9B_R G_R}{3B_R + G_R}. \quad (6)$$

The Hill bulk (B_H), the shear (G_H) modulus and the Young modulus (E_H) are given by averaging the corresponding Voigt and Reuss moduli[6]:

$$B_H = \frac{1}{2}(B_V + B_R) \quad (7)$$

$$G_H = \frac{1}{2}(G_V + G_R) \quad (8)$$

$$E_H = \frac{1}{2}(E_V + E_R),, \quad (9)$$

and the Poisson's ratio (ν_H) is are written as:

$$\nu_H = \frac{3B_H - 2G_H}{2(3B_H + G_H)}. \quad (10)$$

The anisotropy of the material is calculated in terms of the universal elastic anisotropy index (A^U), obtained from the model proposed by Ranganathan and Ostoja-Starzewski[8]:

$$A^U = 5\frac{G_V}{G_R} + \frac{B_V}{B_R} - 6 \geq 0. \quad (11)$$

1.2 Thermodynamic properties

The *P*–*V* equations of state were obtained from the structural relaxation process under pressure ranging from zero to 8.0 GPa, through the QE code. In addition, the heat capacity at constant volume as a function of temperature (T) was studied using a post-processing performed by the Thermo_pw package[1], following the equation:

$$C_V = 9nk_B \left(\frac{T}{\Theta_D}\right)^3 \int_0^{\Theta_D/T} \frac{x^4 e^x}{(e^x - 1)^2} dx, \quad (12)$$

where n is the number of atoms per cell, k_B is Boltzmann's constant, and Θ_D is the Debye temperature. Following Anderson[9], Θ_D was determined from the elastic constant data of the material as shown bellow

$$\Theta_D = \frac{h}{k_B}\left(\frac{3n}{4\pi}\frac{N_A\rho}{M}\right)^{1/3} v_{av}, \tag{13}$$

where h is the Planck constant, N_A is the Avogadro number, ρ is the material density, M is the molecular weight of the solid, and v_{av} is the average sound velocity, which depends on the mechanical properties. The Voigt–Reuss–Hill average of the bulk and shear moduli are necessary to calculate v_{av}. The Debye temperature calculated in this way evaluates the average sound velocity from the angular average of the sound velocities calculated for each propagation direction. Thus, the longitudinal and transverse sound velocities, v_ℓ and v_t, respectively, were obtained from the Hill's moduli[7,10] as:

$$v_\ell = \sqrt{\frac{B_H + 4G_H/3}{\rho}}, \tag{14}$$

$$v_t = \sqrt{\frac{G_H}{\rho}}, \tag{15}$$

and the average sound velocity, v_{av}, which appears in equation 13, was determined from v_ℓ and v_t as follows:

$$v_{av} = \left[\frac{1}{3}\left(\frac{1}{v_\ell^3} + \frac{2}{v_t^3}\right)\right]^{-1/3}. \tag{16}$$

Finally, the exact Debye temperature is used within the Debye model to calculate, for example, the isochoric heat capacity. Furthermore, the Grüneisen acoustic constant (γ) was also calculated as a function of the longitudinal and transverse sound velocities, as proposed by Belomestnykh[11]:

$$\gamma = \frac{9}{2}\frac{v_\ell^2 - 4v_t^2/3}{v_\ell^2 + 2v_t^2}. \tag{17}$$

1.2.1 Lattice thermal conductivity

For defect-free crystals and lattice thermal resistance resulting only from intrinsic phonon-phonon interactions, the Slack model[12,13] has been widely used to calculate the lattice

thermal conductivity (κ_L)[14–17], which is written as:

$$\kappa_L = A\frac{M_a \delta n^{1/3} \Theta_D^3}{\gamma^2 T}, \tag{18}$$

where, for κ_L in units of watt per meter per kelvin,

$$A = \frac{2.43 \times 10^{-6}}{1 - \frac{0.514}{\gamma} + \frac{0.228}{\gamma^2}}, \tag{19}$$

M_a is the average atomic weight of all the constituent atoms, δ^3 is the volume of the primitive unit cell per atom, n is the number of atoms in the primitive unit cell, Θ_D is the Debye acoustic temperature, and γ is the Grüneisen parameter. Both parameters Θ_D and γ can be obtained from lattice dynamic calculations or experimental measurements[12,16]. However, instead of this procedure, Xia *et al.*[18] recently successfully calculated the lattice thermal conductivity using Θ_D and γ given by equations 13 and 17, respectively. In the present work, κ_L for the pristine lizardite ($Mg_3(Si_2O_5)(OH)_4$) was obtained from the Slack model (equation 18), with Θ_D and γ from equations 13 and 17, in line with Xia *et al.* approach[18].

For lizardite with vacancies, the lattice thermal conductivity calculation follows the seminal works of Klemens[19,20] for the effect of point defects in thermal resistance, corroborated by Callaway *et al.*[21] and Abeles[22]. Thus, for lizardite with an oxygen vacancy type, $V_\alpha^\times$, the lattice thermal conductivity, $\kappa_{V_\alpha^\times}$, was determined by:

$$\kappa_{V_\alpha^\times} = \kappa_L \frac{\tan^{-1} u}{u}, \tag{20}$$

where κ_L is the lattice thermal conductivity of the pristine lizardite given by equation 18,

$$u^2 = \frac{\pi^2}{h v_{av}^2} \Theta_D \Omega \kappa_L \Gamma, \tag{21}$$

Ω is the cell volume per atom, h is the Planck's constant, Θ_D and v_{av} are given by equations 13

and 16, respectively, and Γ is written as:

$$\Gamma = \sum_i C_i \frac{(M_i - M)^2}{M^2}, \tag{22}$$

with

$$M = \sum_i C_i M_i, \tag{23}$$

where C_i (M_i) is the concentration (mass) of the atom type i.

The minimum lattice thermal conductivity of lizardite with oxygen vacancy ($\kappa_{V_\alpha^\times (\mathrm{min})}$) is obtained from equation 20, using the minimum value of κ_L for pristine lizardite ($\kappa_{L(\mathrm{min})}$), according to Clarke[23]:

$$\kappa_{L(\mathrm{min})} = 0.87\kappa_\mathrm{B} N_\mathrm{A}^{2/3} \frac{m^{2/3}\rho^{1/6}E_H^{1/2}}{M^{2/3}}. \tag{24}$$

In Table S1 we present the accuracy of the Belomestnykh approximation in relation to the DFT calculation to deterfmine the Grüneisen parameters, using some examples from literature for structure simpler than the lizardite one. It is observed that the results from the Belomestnykh approach are in reasonable agreement with the DFT calculations.

Table: S1 Comparision of the Grüneisen parameters obtained from the Belomestnykh approximation with the DFT calculation for some compounds.

Compound	γ (DFT)	γ(Belomestnykh)
Mg_3Sb_2	1.83[(a)]	1.85[(a)]
$CaMg_2Sb_2$	1.44[(a)]	1.40[(a)]
$CaMg_2Bi_2$	1.46[(a)]	1.45[(a)]
Bi_2Te_3	1.52[(b)]	1.65[(c)]
SnSe	2.83[(d)]	3.13[(d)]
PbTe	1.49[(d)]	1.65[(d)]
PbSe	2.66[(d)]	1.69[(d)]
PbS	2.46[(d)]	1.67[(d)]

(a) Ref.[24]
(b) Ref.[25]
(c) Ref.[26]
(d) Ref.[18]

Table S2: The main structural parameters: lattice type; *a*, *b*, and *c* lattice parameters; α, β, and γ angles; volume; density; and interlayer distance, of the pristine $Mg_3Si_2O_5(OH)_4$ and vacancy-types lizardite. In parentheses, we have added the experimental structural parameters of the pristine lizardite from Mellini[27].

Compound	Lattice type	Lattice parameters (Å)			Angle (°)			Volume (Å^3)	Density (g/cm^3)	Interlayer distance (Å)
		a	*b*	*c*	α	β	γ			
Pristine	Trigonal	5.276	5.276	7.117	90.000	90.000	120.000	171.572	2.682	1.783
	Trigonal	(5.332)	(5.332)	(7.233)	(90.000)	(90.000)	(120.000)	(178.086)	(2.584)	(–)
$V_{O1}^{\times}$	Triclinic	5.103	5.103	7.088	89.497	89.497	117.756	163.292	2.655	1.741
$V_{O2}^{\times}$	Trigonal	5.291	5.291	6.889	90.000	90.000	120.000	166.999	2.596	1.678
$V_{O3}^{\times}$	Triclinic	5.285	5.295	7.103	89.732	90.536	119.941	172.248	2.507	1.780
$V_{O1\text{-}O2}^{\times}$	Triclinic	5.161	5.173	6.829	90.135	90.003	118.268	160.514	2.536	1.614
$V_{O1\text{-}O3}^{\times}$	Triclinic	5.149	5.120	7.177	76.634	94.847	117.898	162.647	2.492	1.656
$V_{O2\text{-}O3}^{\times}$	Triclinic	5.285	5.294	6.892	89.669	89.681	120.012	166.858	2.429	1.640

2 Results

2.1 Structural parameters

The convergence analysis of the total energy with the kinetic energy cutoff and k-mesh, is presented in Fig. S1, as well as the convergence of cell parameter "a" and the cell volume as a function of the energy cutoff Ecutwfc and the k-mesh ($k_x = k_y = k_z$) are shown in figures S2 and S3, respectively, for pristine lizardite and three types of characteristic vacancies.

In the Table S2 are shown the main structural parameters: lattice type, lattice parameters (*a*, *b*, *c*), angles (α, β, γ), volume, density, and interlayer distance, resulting from the structural optimization performed for the pristine and vacancy-types lizardite ($V_{O1}^{\times}$), $V_{O2}^{\times}$, $V_{O3}^{\times}$, $V_{O1\text{-}O2}^{\times}$, $V_{O1\text{-}O3}^{\times}$, and $V_{O2\text{-}O3}^{\times}$).

2.2 Mechanical properties

The elastic stiffness constants c_{ij} and the elastic compliances constants s_{ij}, obtained from the Thermo_pw package[1], are presented in Table S3. We have observed that both matrices $[c_{ij}]$ and $[s_{ij}]$ obey the stability criterion, whereby their eigenvalues must all be greater than zero[2–5]. For pristine lizardite, our results are in good agreement with those obtained by Deng et al.[28] desnity functional theory calculations within the local density approximation (LDA) and generalized

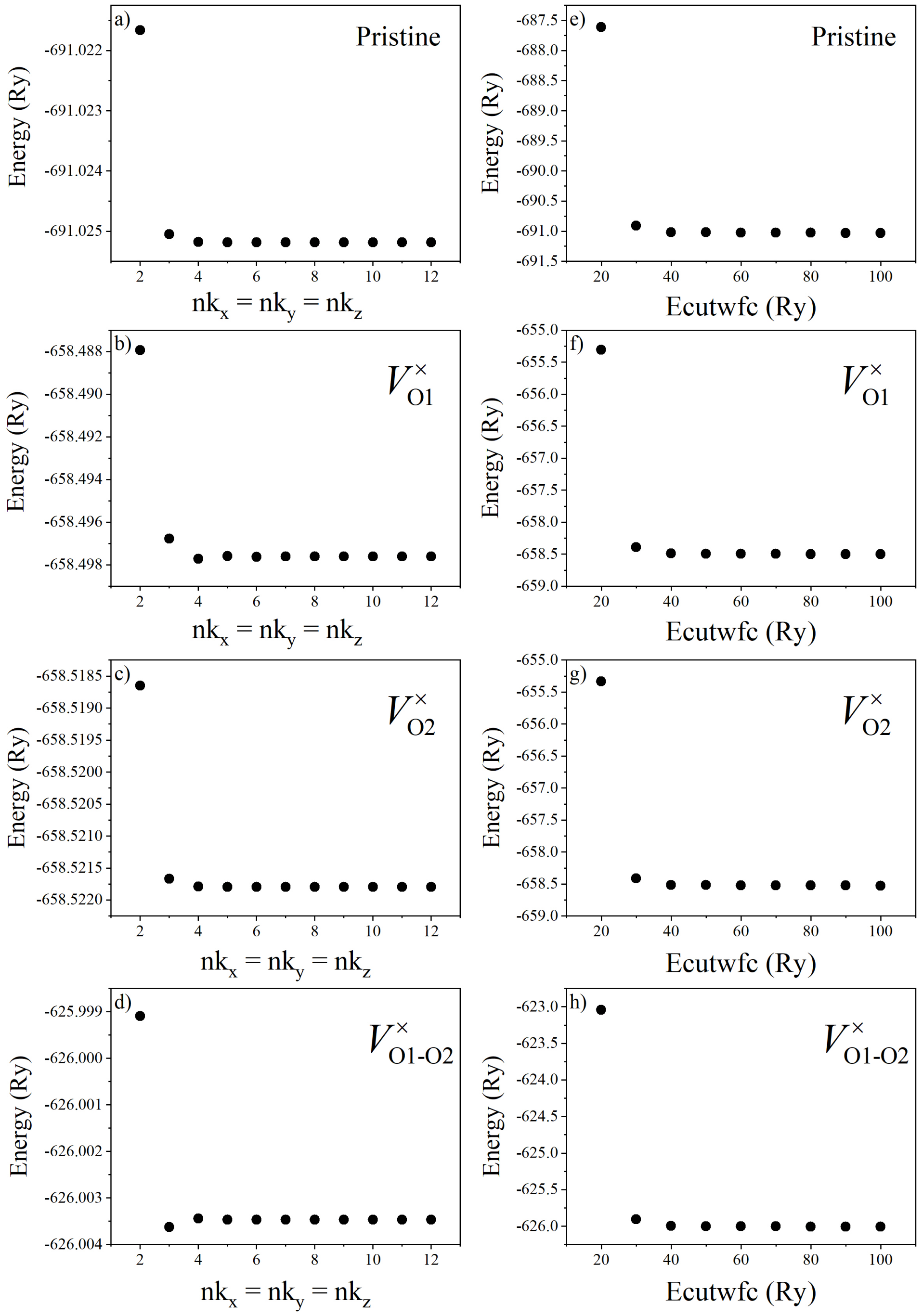

Fig. S1: Convergence of the total energy with the kinetic energy cutoff and k-mesh for pristine lizardite and three types of characteristic vacancies.

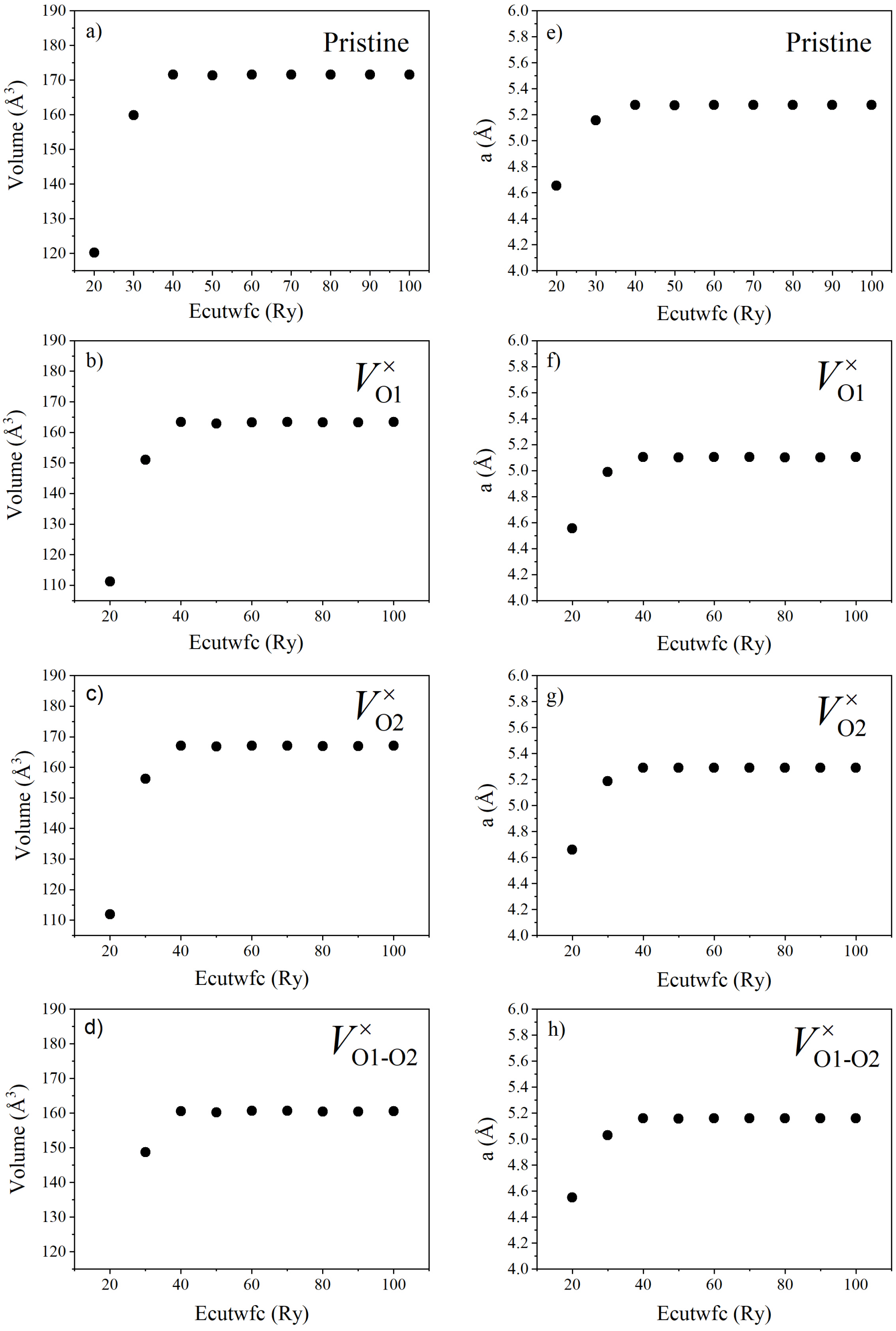

Fig. S2: Convergence of the cell parameter "a" and the cell volume as a function of the energy cutoff Ecutwfc for pristine lizardite and three types of characteristic vacancies.

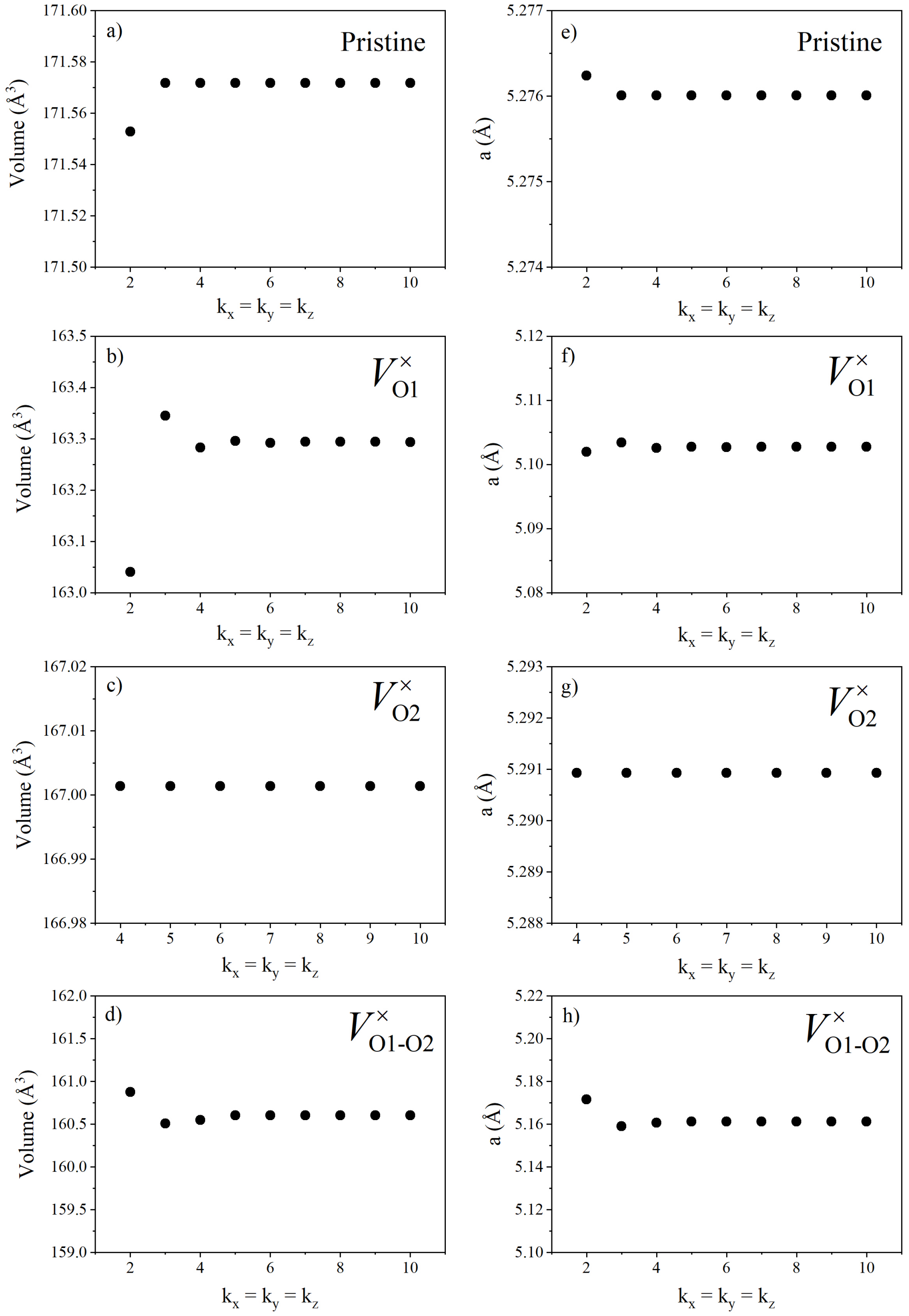

Fig. S3: Convergence of the cell parameter "a" and the cell volume as a function of k mesh ($k_x = k_y = k_z$) for pristine lizardite and three types of characteristic vacancies.

Table S3: Elastic stiffness constants c_{ij} (in GPa) and elastic compliances constants s_{ij} (in 1/Mbar=1/100GPa) of $Mg_3Si_2O_5(OH)_4$ with and without oxygen vacancies.

Compound	c_{11} (s_{11})	c_{12} (s_{12})	c_{13} (s_{13})	c_{22} (s_{22})	c_{23} (s_{23})	c_{33} (s_{33})	c_{44} (s_{44})	c_{55} (s_{55})	c_{66} (s_{66})
Pristine	235.00 (0.507)	87.56 (-0.181)	33.41 (-0.079)	235.08 (0.507)	33.41 (-0.079)	137.64 (0.765)	20.62 (4.898)	20.64 (4.917)	74.90 (1.348)
Pristine (LDA)[(a)]	227.33	82.28	27.27	227.33	27.27	124.74	16.24	16.24	73.02
Pristine (GGA)[(a)]	215.72	74.22	8.11	215.72	8.11	59.68	10.63	10.63	70.50
Pristine (LDA)[(b)]	235.61	85.96	25.05	235.61	25.05	118.16	20.92	20.92	74.83
Pristine (GGA)[(c)]	212.26	73.30	8.50	212.26	8.50	57.30	11.60	11.60	69.70
$V_{O1}^{\times}$	188.26 (0.602)	63.29 (-0.184)	18.99 (-0.072)	209.27 (0.552)	20.43 (-0.065)	114.43 (0.929)	12.70 (8.190)	12.55 (8.149)	77.76 (1.327)
$V_{O2}^{\times}$	214.71 (0.552)	80.18 (-0.193)	34.70 (-0.083)	214.75 (0.552)	34.72 (-0.083)	149.48 (0.708)	20.51 (4.891)	21.05 (4.762)	69.37 (1.446)
$V_{O3}^{\times}$	176.28 (0.646)	53.56 (-0.179)	25.96 (-0.142)	176.82 (0.631)	19.81 (-0.100)	85.85 (1.304)	8.61 (11.660)	13.51 (7.901)	63.48 (1.581)
$V_{O1\text{-}O2}^{\times}$	149.16 (0.768)	49.14 (-0.189)	23.42 (-0.121)	178.79 (0.649)	29.39 (-0.113)	125.37 (0.885)	17.78 (5.844)	15.47 (6.885)	63.41 (1.656)
$V_{O1\text{-}O3}^{\times}$	100.78 (1.274)	38.27 (-0.414)	31.61 (-0.173)	126.55 (1.158)	23.99 (-0.255)	101.88 (1.385)	14.85 (8.180)	9.22 (16.636)	55.45 (2.133)
$V_{O2\text{-}O3}^{\times}$	173.58 (0.659)	59.53 (-0.209)	22.09 (-0.084)	176.46 (0.665)	30.07 (-0.147)	99.63 (1.121)	14.75 (7.035)	11.63 (8.977)	61.52 (1.641)

(a) Ref.[28]
(b) Ref.[29]
(c) Ref.[30]

gradient approximation (GGA), Mookherjee and Stixrude[29] using LDA, and Tsuchiya[30], using GGA.

2.3 Lattice thermal conductivity

In Fig. S1 is shown the lattice thermal conductivity calculated in the quasi-harmonic approximation for pristine lizardite. We observed that, around the Debye temperature, for which the Belomestnykh approximation is applied, that result is in good agreement with the Belomestnykh estimation.

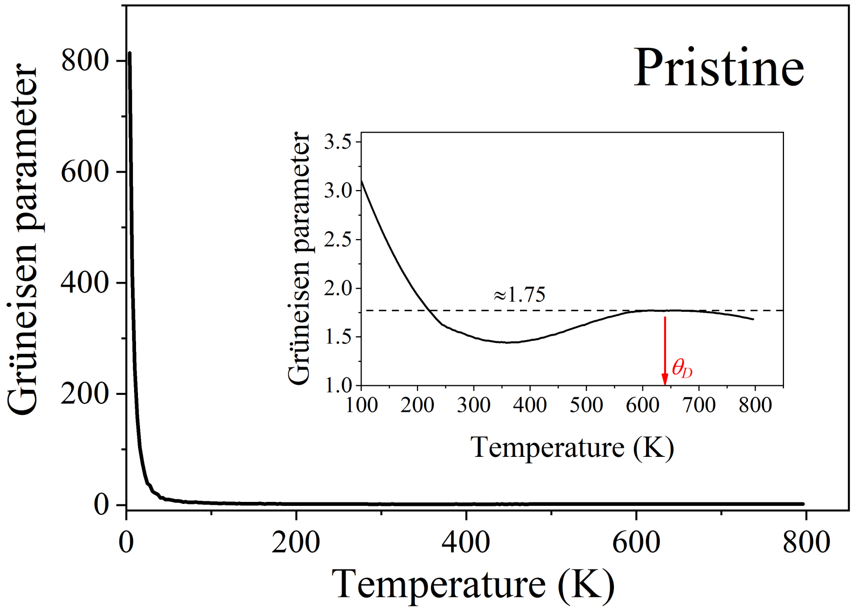

Figure S1: Fig. S4: Lattice thermal conductivity calculated in the quasi-harmonic approximation using the *Thermo_pw* package for pristine lizardite.

3 Atomic positions in crystal coordinates

Below, we provide the atomic coordinates (*xyz* positions).

Pristine lizardite

Atom	x	y	z
Si	0.3333333430	0.6666666870	0.0726218751
Si	0.6666666870	0.3333333430	0.0726218751
Mg	0.3307130093	-0.0000000000	0.4620479558
Mg	0.0000000000	0.3307130093	0.4620479558
Mg	0.6692869907	0.6692869907	0.4620479558
O	0.3333333430	0.6666666870	0.2964197472
O	0.6666666870	0.3333333430	0.2964197472
O	0.5223130125	0.0000000000	0.9844624071
O	-0.0000000000	0.5223130125	0.9844624071
O	0.4776869875	0.4776869875	0.9844624071
O	0.6652204715	-0.0000000000	0.5971205817
O	-0.0000000000	0.6652204715	0.5971205817
O	0.3347795285	0.3347795285	0.5971205817
O	-0.0000000000	-0.0000000000	0.3011675239
H	0.6471662848	-0.0000000000	0.7339647528
H	0.0000000000	0.6471662848	0.7339647528
H	0.3528337152	0.3528337152	0.7339647528
H	-0.0000000000	-0.0000000000	0.1644622040

Vacancy type $V_{O1}^{\times}$

Atom	x	y	z
Si	0.3582996086	0.6611494410	0.0686973178
Si	0.6611494400	0.3582996092	0.0686973175
Mg	0.3321234212	-0.0006879192	0.4569698701
Mg	-0.0006879190	0.3321234202	0.4569698697
Mg	0.6689669909	0.6689669912	0.4683099220
O	0.3396615487	0.6668623666	0.2947799521
O	0.6668623665	0.3396615490	0.2947799518
O	0.5145023265	0.0117707877	0.9824122937
O	0.0117707871	0.5145023276	0.9824122930
O	0.6689051197	0.0061294564	0.6004486953
O	0.0061294567	0.6689051175	0.6004486978
O	0.3330187996	0.3330187993	0.6035456452
O	0.0001360420	0.0001360423	0.2989121836
H	0.6404580265	0.0141233201	0.7367056665
H	0.0141233174	0.6404580248	0.7367056625
H	0.3055541601	0.3055541598	0.7395302034
H	0.0013395801	0.0013395789	0.1617121157

Vacancy type $V_{O2}^{\times}$

Atom	x	y	z
Si	0.3333333430	0.6666666870	0.0987382766
Si	0.6666666870	0.3333333430	0.0630351068
Mg	0.3319752410	0.0108702916	0.4629624232
Mg	-0.0108702916	0.3211049494	0.4629624232
Mg	0.6788950506	0.6680247590	0.4629624232
O	0.6666666870	0.3333333430	0.2970212333
O	0.5171403649	-0.0039219471	0.9810499506
O	0.0039219471	0.5210623120	0.9810499506
O	0.4789376880	0.4828596351	0.9810499506
O	0.6559905745	-0.0111000224	0.5956718808
O	0.0111000224	0.6670905968	0.5956718808
O	0.3329094032	0.3440094255	0.5956718808
O	-0.0000000000	-0.0000000000	0.2958732715
H	0.6226938571	-0.0243508619	0.7374711589
H	0.0243508619	0.6470447190	0.7374711589
H	0.3529552810	0.3773061429	0.7374711589
H	0.0000000000	0.0000000000	0.1543802583

Vacancy type $V_{O3}^{\times}$

Atom	x	y	z
Si	0.3327001027	0.6688617054	0.0712181840
Si	0.6638384283	0.3311383246	0.0712181840
Mg	0.3298239388	-0.0000000000	0.4593068413
Mg	0.0086822372	0.3368217459	0.4580133580
Mg	0.6718604912	0.6631782541	0.4580133580
O	0.3362654794	0.6677691232	0.2981995689
O	0.6684963872	0.3322309068	0.2981995689
O	0.5163692450	-0.0000000000	0.9872062938
O	-0.0029908490	0.5222386882	0.9863254008
O	0.4747704629	0.4777613118	0.9863254008
O	0.0050900874	0.6649579009	0.5978506800
O	0.3401321864	0.3350420991	0.5978506800
O	-0.0021510780	-0.0000000000	0.3051604194
H	0.0036359606	0.6479771070	0.7356784542
H	0.3556588536	0.3520228930	0.7356784542
H	-0.0136805156	-0.0000000000	0.1673569988

Vacancy type $V_{O1\text{-}O2}^{\times}$

Atom	x	y	z
Si	0.4433609159	0.7612164373	0.1965512953
Si	0.7506636781	0.4507066862	0.1549191498
Mg	0.4353517877	0.1097685410	0.5592959229
Mg	0.0878796757	0.4202435155	0.5607015788
Mg	0.7785767851	0.7679326171	0.5644102423
O	0.7647397657	0.4346600530	0.3939864084
O	0.6139470653	0.0947671591	1.0802336586
O	0.1076828589	0.6071993276	1.0799814596
O	0.7635161634	0.0945838350	0.7000640150
O	0.1186756289	0.7721218214	0.6968025583
O	0.4370464110	0.4451373368	0.7007676227
O	0.1006721907	0.0980631438	0.3922113855
H	0.7241686813	0.0853085106	0.8426354773
H	0.1332672475	0.7462014664	0.8392790837
H	0.4388636632	0.4628799187	0.8439396767
H	0.0905672111	0.1048560161	0.2498383757

Vacancy type $V^{\times}_{O1\text{-}O3}$

Atom	x	y	z
Si	0.3660347512	0.7442027302	0.0615619424
Si	0.6647435046	0.4443501283	0.0659503459
Mg	0.3216717137	-0.0354797814	0.4486198426
Mg	0.0072641235	0.3049885606	0.4607566243
Mg	0.6625340103	0.6216323101	0.4599495595
O	0.3416677804	0.6827437430	0.2961630110
O	0.6703548535	0.3426599762	0.3000021921
O	0.4997650868	0.1232260544	0.9818482988
O	0.0168097097	0.5999729900	0.9840446005
O	-0.0038167447	0.5881179469	0.6026912881
O	0.3286544007	0.2447218647	0.6055570252
O	-0.0088077253	0.0133818394	0.3037589913
H	0.0032284408	0.5670124503	0.7430761086
H	0.3532366019	0.1909397653	0.7445549527
H	-0.0187289311	0.0836396402	0.1658429780

Vacancy type $V^{\times}_{O2\text{-}O3}$

Atom	x	y	z
Si	0.4378972686	0.7842165991	0.1964980591
Si	0.7693803284	0.4483475895	0.1626326001
Mg	0.4289142459	0.0875945992	0.5475166280
Mg	0.0879627856	0.4166253912	0.5588913814
Mg	0.7718207170	0.7592880097	0.5675083696
O	0.7669609974	0.4322565686	0.3985519303
O	0.6212101708	0.1143230237	1.0776189894
O	0.1086008751	0.6363978037	1.0823149824
O	0.5900288767	0.6067049820	1.0885306875
O	0.7529840064	0.0848992869	0.6973662492
O	0.1077759052	0.7591134876	0.6972478522
O	0.0926915799	0.0917667141	0.3985919947
H	0.7184653779	0.0808545953	0.8394612693
H	0.1254873823	0.7432136470	0.8395906927
H	0.0988729560	0.1001178317	0.2566732970

References

1 For information about the thermo_pw software:. `https://dalcorso.github.io/thermo\_pw/`.

2 Bower, A. F. *Applied Mechanics of Solids*; CRC Press, 2010.

3 Born, M. On the stability of crystal lattices. I. Mathematical Proceedings of the Cambridge Philosophical Society. 1940; pp 160–172.

4 Mouhat, F.; Coudert, F.-X. Necessary and sufficient elastic stability conditions in various crystal systems. *Phys. Rev. B* **2014**, *90*, 224104.

5 Nye, J. F. *Physical properties of crystals: their representation by tensors and matrices*; Oxford university press, 1985.

6 Hill, R. The elastic behaviour of a crystalline aggregate. *Proc. Phys. Soc. A* **1952**, *65*, 349.

7 Love, A. E. H. *A treatise on the mathematical theory of elasticity*; Cambridge university press, 2013.

8 Ranganathan, S. I.; Ostoja-Starzewski, M. Universal elastic anisotropy index. *Phys. Rev. Lett.* **2008**, *101*, 055504.

9 Anderson, O. L. A simplified method for calculating the Debye temperature from elastic constants. *J. Phys. Chem. Solids* **1963**, *24*, 909–917.

10 Birch, F. The velocity of compressional waves in rocks to 10 kilobars: 1. *J. Geophys. Res.* **1960**, *65*, 1083–1102.

11 Belomestnykh, V. N. The acoustical Grüneisen constants of solids. *Tech. Phys. Lett.* **2004**, *30*, 91–93.

12 Slack, G. In *Solid State Physics: Advances in Research and Applications*; Ehrenreich, H., Seitz, F., Turnbull, D., Eds.; Academic Press, New York, 1979; Vol. 34; p 1.

13 Slack, G. A. Nonmetallic crystals with high thermal conductivity. *J. Phys. Chem. Solids* **1973**, *34*, 321–335.

14 Morelli, D.; Jovovic, V.; Heremans, J. Intrinsically minimal thermal conductivity in cubic I – V – VI 2 semiconductors. *Phys. Rev. Lett.* **2008**, *101*, 035901.

15 Skoug, E. J.; Cain, J. D.; Morelli, D. T. Structural effects on the lattice thermal conductivity of ternary antimony-and bismuth-containing chalcogenide semiconductors. *Appl. Phys. Lett.* **2010**, *96*, 181905.

16 Shindé, S. L.; Goela, J. *High thermal conductivity materials*; Springer, 2006; Vol. 91.

17 Nielsen, M. D.; Ozolins, V.; Heremans, J. P. Lone pair electrons minimize lattice thermal conductivity. *Energy Environ. Sci.* **2013**, *6*, 570–578.

18 Xiao, Y.; Chang, C.; Pei, Y.; Wu, D.; Peng, K.; Zhou, X.; Gong, S.; He, J.; Zhang, Y.; Zeng, Z., et al. Origin of low thermal conductivity in SnSe. *Phys. Rev. B* **2016**, *94*, 125203.

19 Klemens, P. The scattering of low-frequency lattice waves by static imperfections. *Proc. Phys. Soc. A* **1955**, *68*, 1113.

20 Klemens, P. Thermal resistance due to point defects at high temperatures. *Phys. Rev.* **1960**, *119*, 507.

21 Callaway, J.; von Baeyer, H. C. Effect of point imperfections on lattice thermal conductivity. *Phys. Rev.* **1960**, *120*, 1149.

22 Abeles, B. Lattice thermal conductivity of disordered semiconductor alloys at high temperatures. *Phys. Rev.* **1963**, *131*, 1906.

23 Clarke, D. R. Materials selection guidelines for low thermal conductivity thermal barrier coatings. *Surf. Coat. Technol.* **2003**, *163*, 67–74.

24 Peng, W.; Petretto, G.; Rignanese, G.-M.; Hautier, G.; Zevalkink, A. An unlikely route to low lattice thermal conductivity: small atoms in a simple layered structure. *Joule* **2018**, *2*, 1879–1893.

25 Park, K.; Mohamed, M.; Aksamija, Z.; Ravaioli, U. Ab initio lattice dynamics and thermochemistry of layered bismuth telluride (Bi2Te3). *J Appl Phys* **2015**, *117*, 015103.

26 Peng, W.; Smiadak, D. M.; Boehlert, M. G.; Mather, S.; Williams, J. B.; Morelli, D. T.; Zevalkink, A. Lattice hardening due to vacancy diffusion in (GeTe) mSb2Te3 alloys. *Journal of Applied Physics* **2019**, *126*.

27 Mellini, M. The crystal structure of lizardite 1T: hydrogen bonds and polytypism. *Am. Mineral.* **1982**, *67*, 587–598.

28 Deng, X.; Luo, C.; Wentzcovitch, R. M.; Abers, G. A.; Wu, Z. Elastic anisotropy of lizardite at subduction zone conditions. *Geophys. Res. Lett.* **2022**, *49*, e2022GL099712.

29 Mookherjee, M.; Stixrude, L. Structure and elasticity of serpentine at high-pressure. *Earth Planet. Sci. Lett.* **2009**, *279*, 11–19.

30 Tsuchiya, J. A first-principles calculation of the elastic and vibrational anomalies of lizardite under pressure. *Am. Mineral.* **2013**, *98*, 2046–2052.
